# Preconditioning with Triiodothyronine Improves the Clinical Signs and Acute Tubular Necrosis Induced by Ischemia/Reperfusion in Rats

**DOI:** 10.1371/journal.pone.0074960

**Published:** 2013-09-26

**Authors:** Carla Ferreyra, Félix Vargas, Isabel Rodríguez-Gómez, Rocío Pérez-Abud, Francisco O'Valle, Antonio Osuna

**Affiliations:** 1 Servicio de Nefrología, Unidad Experimental, Hospital Virgen de las Nieves, Granada, Spain; 2 Departamento de Fisiología, Facultad de Medicina, Granada, Spain; 3 Departamento de Anatomía Patológica e Instituto de Biomedicina Regenerativa, Facultad de Medicina, Granada, Spain; National Cancer Institute, United States of America

## Abstract

**Background:**

Renal ischemia/reperfusion (I/R) injury is manifested by acute renal failure (ARF) and acute tubular necrosis (ATN). The aim of this study was to evaluate the effectiveness of preconditioning with 3, 3, 5 triiodothyronine (T_3_) to prevent I/R renal injury.

**Methodology/Principal Findings:**

The rats were divided into four groups: sham-operated, placebo-treated (SO-P), sham-operated T_3-_ treated (SO- T_3_), I/R-injured placebo-treated (IR-P), and I/R-injured T_3_-treated (IR- T_3_) groups. At 24 h before ischemia, the animals received a single dose of T_3_ (100 μg/kg). Renal function and plasma, urinary, and tissue variables were studied at 4, 24, and 48 h of reperfusion, including biochemical, oxidative stress, and inflammation variables, PARP-1 immunohistochemical expression, and ATN morphology. In comparison to the SO groups, the IR-P groups had higher plasma urea and creatinine levels and greater proteinuria (at all reperfusion times) and also showed: increased oxidative stress-related plasma, urinary, and tissue variables; higher plasma levels of IL6 (proinflammatory cytokine); increased glomerular and tubular nuclear PARP-1 expression; and a greater degree of ATN. The IR-T_3_ group showed a marked reduction in all of these variables, especially at 48 h of reperfusion. No significant differences were observed between SO-P and SO-T_3_ groups.

**Conclusions:**

This study demonstrates that preconditioning rats with a single dose of T_3_ improves the clinical signs and ATN of renal I/R injury. These beneficial effects are accompanied by reductions in oxidative stress, inflammation, and renal PARP-1 expression, indicating that this sequence of factors plays an important role in the ATN induced by I/R injury.

## Introduction

Renal ischemia/reperfusion (I/R) injury is a major cause of acute renal failure (ARF), which can manifest histologically as acute tubular necrosis (ATN) [1]. It can result from systemic hypoperfusion or from the temporary interruption of renal blood supply in clinical procedures such as kidney transplantation, partial nephrectomy, renal artery angioplasty, aortic aneurysm surgery, and elective urological surgery, among others.

High concentrations of reactive oxygen species (ROS) are generated in ischemic organs after reperfusion. During I/R injury (and similar conditions), the increase in oxidative stress can damage cellular components such as DNA, proteins, and lipids [2]–[4], thereby directly compromising the integrity of the glomerular and tubular epithelium, an event known to contribute to the development of ATN [5].

In the 1970s, Straub et al. [6], [7] demonstrated that the administration of thyroxine (T_4_) in animals (mice and rabbits) with nephrotoxic renal failure achieved a marked reduction in their mortality rate. Subsequent studies of rats with nephrotoxic ARF induced by various nephrotoxic agents found that T_4_ improved the renal morphology by accelerating the repair of injured renal tubules, leading to a more rapid recovery of renal function [8]–[11]. In an *in vitro* study, Johnson et al. [12] observed that pre-treatment of rabbit proximal tubular cells with 3, 3, 5 triiodothyronine (T_3_) increased their response to epidermal growth factor and accelerated tubular regeneration. In “in vivo” studies, it was reported that post-ischemic T_4_ administration improved the renal function and cellular morphology, with a greater recovery of the intracellular renal ATP content, which was depleted by ischemic ARF [13]. Pre-treatment with T_3_ was also found to protect the liver against I/R injury in rats [14].

The deleterious effects of I/R injury are triggered by a complex response involving oxygen radical species, cytokines, and chemokines [15]–[17]. Previous studies by our group showed that I/R injury causes poly (ADP-ribose) polymerase-1 (PARP-1) overexpression, which is associated with a high incidence of ATN and delayed graft function [18]. Moreover, kidney I/R injury is known to engage cellular mediators of immunity, such as dendritic cells, neutrophils, macrophages, natural killer T, T, and B cells, which contribute to the pathogenesis of the renal injury [19]. Thus, leucocyte-depleted hemoreperfusion improved post-ischemic renal function and tubulointerstitial damage in a porcine model [20], and it has been shown that the immune response and, more specifically, lymphocytes (T and B) and dendritic cells participate as mediators of renal I/R injury [21].

With this background, we designed a study to test the hypothesis that treatment with T_3_ before I/R can protect the kidney against I/R injury by correcting the associated imbalance in oxidative status. For this purpose, the objective of this study was to assess the effects of preconditioning with T_3_ on renal function, oxidative stress, inflammatory cytokines, PARP-1 expression, ATN, and leukocyte infiltration in I/R injury.

## Methods

### Animals

Male Wistar rats (n = 120) born and raised in the experimental animal service of the University of Granada were used. Experiments were performed according to European Union guidelines for the ethical care of animals and were approved by the ethical committee of the University of Granada. Rats initially weighing 250–280 g were maintained on standard chow and tap water *ad libitum*. The animals were divided into four groups: IR-T_3,_ rats subjected to bilateral renal ischemia pre-treated with T_3_; IR-P, rats subjected to bilateral renal ischemia pre-treated with placebo; and two groups of rats that underwent a sham laparotomy and were pre-treated with T_3_ (SO-T_3_) or placebo (SO-P). Animals (n = 10 in each group) were examined at 4, 24, or 48 h of reperfusion.

### Experimental protocol

All animals were preconditioned at 24 h before renal ischemia with a single intraperitoneal dose of T_3_ dissolved in 0.1N of NaOH isotonic saline (100 μg/kg body weight) or of the placebo solution (0.1N NaOH isotonic saline, 0.5 ml). Animals were anesthetized by the intraperitoneal injection of equitensin, an anesthetic mixture of pentobarbital, chloral hydrate, dihydroxypropane, and ethanol (0.30 ml/100 g b.w). A polyethylene catheter (PE-50) containing 100 units of heparin in isotonic sterile NaCl solution was inserted into the carotid artery to draw blood samples. The catheter was tunneled subcutaneously and brought out through the skin at the dorsal neck.

The abdomen was shaved and opened through the linea alba to avoid blood losses. Both renal pedicles were identified and occluded with microvascular clamps (Equipamientos sanitarios S.A. Madrid, Spain) for 45 min, after which the clamps were removed, allowing reperfusion of the kidneys. Then, the abdomen was closed in two layers. Occlusion was visually verified by a change in the color of the kidney, which was paler after the occlusion and more bluish after the reperfusion. Sham-operated animals underwent an identical surgical procedure to that of the IR groups, except that the renal pedicles were not clamped (i.e., no occlusion). All rats were housed in meta­bolic cages with food and water *ad libitum* during the reperfusion period (Panlab, Barcelona, Spain), and samples of their urine were collected.

Blood samples were used to determine plasma concentrations of urea, creatinine, malondialdehyde (MDA), glutathione (GSH), and interleukin 6 (IL-6). The urinary variables measured were creatinine, proteinuria, and total isoprostane and hydrogen peroxide (H_2_O_2_) excretions.

At predetermined time points, rats were killed by the injection of sodium pentobarbital and lidocaine hydrochloride and the kidneys were then removed. One kidney was dissected to separate the cortex and medulla and the other was used for the histopathological and immunohistochemical analyses.

### Analytical procedures

Plasma and urinary electrolyte, urea, and creatinine levels were measured with an autoanalyzer (Hitachi-912, Roche, Spain). Proteinuria was determined by using the DC Protein Assay Kit (Bio-Rad, Madrid, Spain). Plasma and renal tissue MDA and GSH concentrations were measured with the TBARS and Glutathione Assay Kits, respectively (Cayman Chemicals Company, USA). Plasma IL6 was measured with an ELISA Kit (R&D systems, Minneapolis, USA). Isoprostanes and H_2_O_2_ in urine were measured by using the 8-Isoprostane EIA Kit and Hydrogen Peroxide Assay Kit, respectively (Cayman Chemicals Company, USA).

### Histopathological study

Kidney samples were fixed in 10% buffered formalin for 24 h and paraffin-embedded, and sections were then stained with hematoxylin/eosin (H/E), Periodic Acid Schiff (PAS), and Masson's trichrome (MT) for morphological study. Histopathological evaluation was done in a blinded fashion (CF and FO) on 4-micrometer sections under light microscopy. The presence of ATN, glomerulitis (presence of more than two leukocytes in some glomeruli), capillaritis (presence of two or more leukocytes in dilated peritubular capillaries, tubulitis (presence of two lymphocytes in tubular cells), sloughing, and the vacuolization of tubular cells were calculated semiquantitatively on a 4-point scale (0, absence; 1, mild [<10% of tubules, capillaries, or glomeruli involved]; 2, moderate [10–25%]; 3, severe [>25%]). The other variables (vascular lesion, glomerular lesion, apoptosis, hyaline globules, altered/lost brush border, tubular cast, and regenerative signs [mitosis and increased basophilia]) were dichotomous (presence/absence).

### Immunohistochemical analysis

Kidney sections were dewaxed, hydrated, and heat-treated in 1 mM EDTA buffer for antigenic unmasking in a PT module (Thermo Fisher Scientific, Kalamazoo, MI) at 95°C for 20 min. Sections were incubated for 30 min at room temperature with PARP-1 polyclonal antibody (Thermo Fisher Scientific), anti-CD45 (clone OX30) (sc-53047 Santa Cruz Biotechnology Inc. Heidelberg, Germany), anti-CD68 (clone ED1) (sc59103 Santa Cruz Biotechnology Inc.), and anti-myeloperoxidase polyclonal antibody (Master Diagnóstica, Granada, Spain). An automatic immunostainer (model autostainer 480, Thermo Fisher Scientific) was used for the immunochemistry study, applying a polymer peroxidase-based method followed by development with diaminobenzidine (Master Diagnóstica). The tubular and glomerular positivity for PARP-1 was scored semi-quantitatively on a 4-point scale (see above). Renal sections incubated with IgG isotype antibody were used as negative controls. A millimetre scale in the eyepiece of a BH2 microscope (Olympus Optical Company, Ltd., Tokyo, Japan) with a 40× objective was used to count the number of inflammatory positive cells per mm^2^.

For the analysis of anti-CD45, CD68, and MPO, immunohistochemistry was used to quantify the number of inflammatory cells (total leukocytes, monocytes/macrophages and granulocytes) per square millimetre in cortical kidney.

### Statistical Analyses

Quantitative plasma, renal, and urinary variables were analyzed with two-way ANOVA design (groups x times) to test for the group x time interaction. When a significant result was obtained, Tukey's “post-hoc” test was used for pairwise comparisons. Data were logarithmically transformed to achieve normality and homogeneity of variances. Because the data were non-normally distributed, the Kuskal-Wallis test was used to detect differences in histological scores and immunohistochemical data among the groups, time by time, followed by pairwise comparisons (Tukey's test) when the result was significant. P<0.05 was considered significant in all tests.

## Results

### Plasma and urinary biochemical variables

As shown in Table 1, plasma creatinine levels were higher in the IR-P than in the SO rats at 4, 24, and 48 h of reperfusion. The T_3_-treated IR group showed a marked reduction in plasma creatinine at 48 h of reperfusion in comparison to the IR-P group, which was similar to that observed in both SO groups (T_3_-treated and -untreated). Similar results were found for the plasma urea levels. Creatinine clearance, according to plasma creatinine values, was lower in the IR-P group than in the SO groups at 4, 24, and 48 h of reperfusion, and the clearance values in the IR-T_3_ group at 48 h of reperfusion were restored to similar levels to those in the SO groups. Proteinuria was higher in both IR groups than in the SO groups at 4, 24, and 48 h of reperfusion but was halved by T_3_ administration at all reperfusion times, although never reaching normal values.

**Table 1:** Variables of renal function.

	Reperfusion period (h)	SO-P	SO-T_3_	IR-P	IR-T_3_
**Plasma Creatinine (mg/dl)**	4	0,28±0,05	0,27±0,03	0,77±0,21**	1,03±0,33† †
	24	0,25±0,05	0,22±0,04	0,45±0,16**	0,62±0,21† †
	48	0,27±0,03	0,26±0,02	0,52±0,06**	0,28±0,04 ‡ ‡
**Plasma Urea** **(mg/dl)**	4	27,9±4,6	31,1±5,6	49,4±4,7**	61,7±7,8† †
	24	24,1±4,8	23,1±3,3	43,2±12,7**	68,1±14,3† †
	48	28,2±3,4	29,6±3,3	42,9±2,2**	35,4±3,6† † ‡
**Proteinuria (mg/ml/100g body wt)**	4	0,43±0,11	0,47±0,11	5,41±0,38**	3,71±0,44† † ‡
	24	0,47±0,09	0,38±0,08	4,22±0,75**	1,81±0,44† † ‡
	48	0,36±0,1	0,32±0,04	2,79±0,26**	1,22±0,13† † ‡
**Creatinine Clearence (ml/min/ 100g body wt)**	4	0,62±0,14	0,54±0,17	0,35±0,31**	0.15±0,13† †
	24	0,78±0,17	0,68±0,14	0,39±0,08**	0,35±0,09† †
	48	0,58±0,08	0,58±0,06	0,22±0,09**	0,57±0,15 ‡ ‡

SO-P = sham-operated-placebo; SO-T_3_ =  sham-operated, T_3_-treated (100 μg/kg); IR-P =  ischemia-reperfusion, placebo-treated; IR-T_3_ =  ischemia-reperfusion, T_3_-treated. Data are means ± SEM, n = 10 each group. * p<0.01, ** p<0.001 compared with the SO-P group. † p<0.01, † † p<0.001 compared with the SO-T_3_ group. ‡ p<0.01, ‡ ‡ p<0.001 compared with the IR-P group.

### Oxidative stress and inflammatory variables

**Figure 1:**
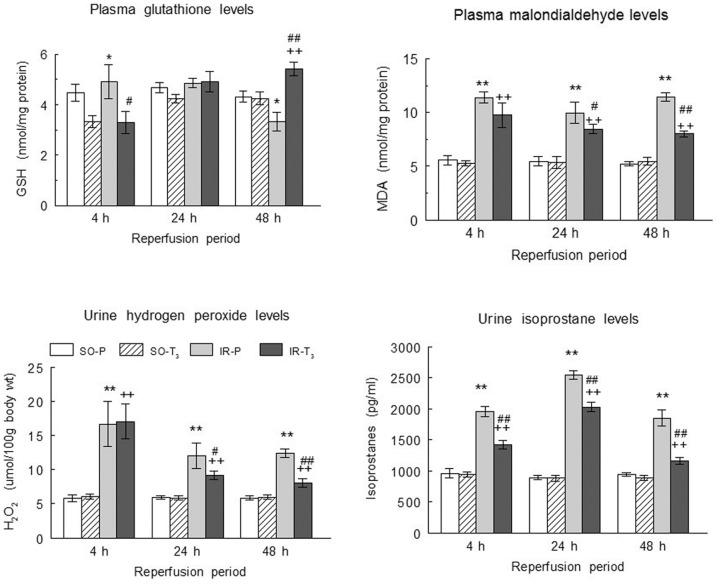
Plasma glutathione and MDA levels, and total urinary excretion of hydrogen peroxide and of isoprostanes. (Upper panels) Plasma glutathione and MDA levels, and (lower panels) total urinary excretion of hydrogen peroxide and of isoprostanes in the experimental groups (n = 10 each group),: SO-P  =  sham-operated-placebo; SO-T_3_ =  sham-operated, T_3_-treated (100 μg/kg); IR-P =  ischemia-reperfusion, placebo-treated; IR-T_3_ =  ischemia-reperfusion, T_3_-treated. Data are means ± SEM. * p<0.05, ** p<0.001 *versus* SO-P group. + p<0.01, ++ p<0.001 *versus* SO-T_3_ group. # p<0.05, ## p<0.001 *versus* IR-P group.

**Figure 2:**
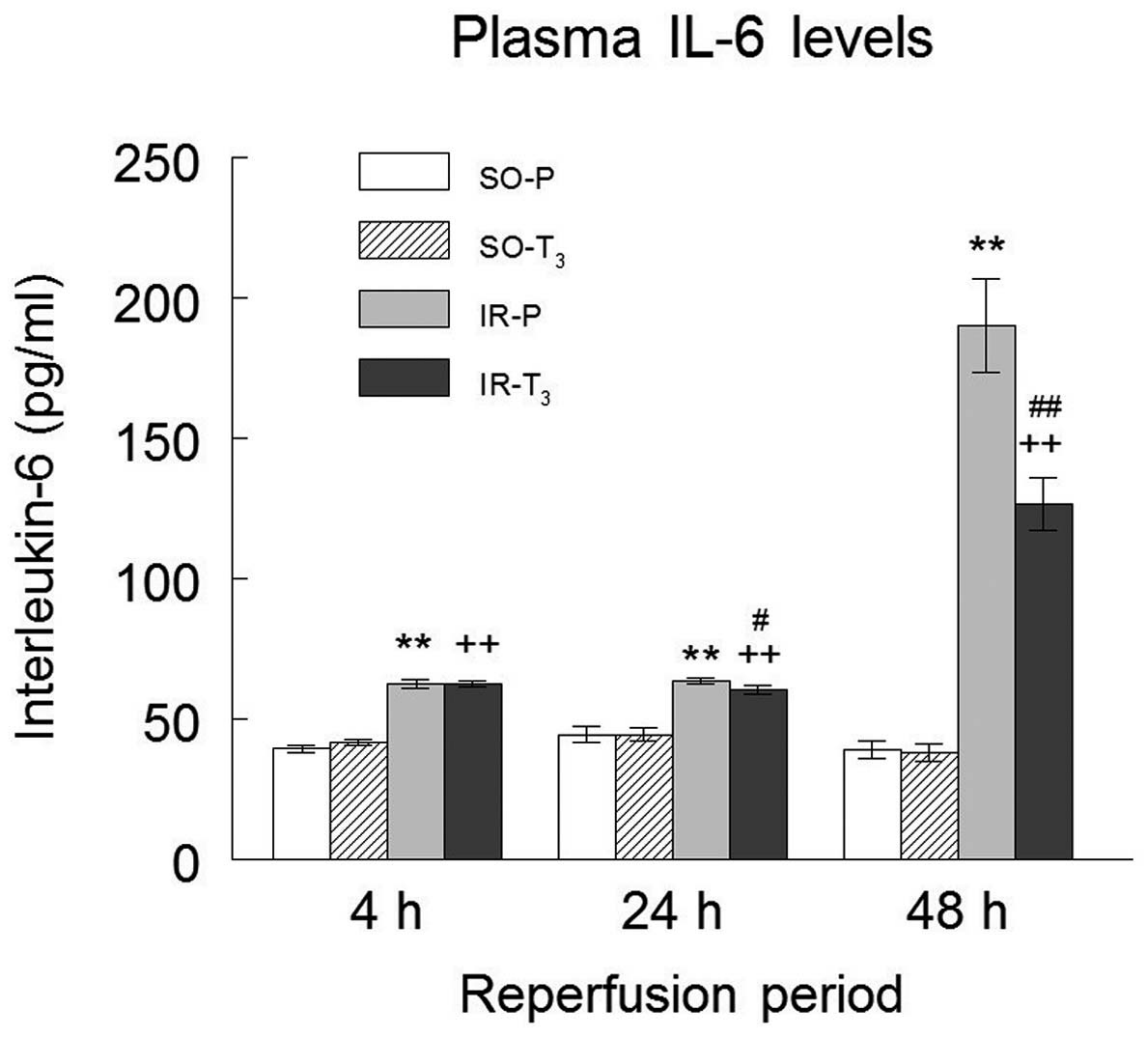
Plasma IL6 levels in the experimental groups. (n = 10 each group): SO-P  =  sham-operated-placebo; SO-T_3_ =  sham-operated, T_3_-treated (100 μg/kg); IR-P =  ischemia-reperfusion, placebo-treated; IR-T_3_ =  ischemia-reperfusion, T_3_-treated. Data are means ± SEM. ** p<0.001 *versus* SO-P group. ++ p<0.001 *versus* SO-T_3_ group. # p<0.05, ## p<0.001 *versus* IR-P group.

Plasma GSH levels were lower in T_3_ groups (SO and IR) than in the SO-P and IR-P groups at 4 h; levels were similar among all groups at 24 h, and they were lower in the IR-P group and higher in the IR-T_3_ group than in either SO group at 48 h of reperfusion (fig 1). Plasma MDA was higher in the IR groups than in the SO groups and was significantly reduced by the T_3_ treatment at all reperfusion times, observing the greatest reduction at 48 h (fig 1). Plasma IL-6 levels were also higher in the IR groups than in the SO groups and were reduced by T_3_ treatment at 24 h and 48 h of reperfusion, with a greater reduction at 48 h (fig 2).

Similar to the findings for the plasma oxidative stress variables, the urinary isoprostane and hydrogen peroxide levels were higher in both IR groups than in both SO groups and were significantly reduced by T_3_ treatment at all reperfusion times, observing the greatest reduction at 48 h, except for the hydrogen peroxide levels, which were not reduced at 4 h (fig 1).

**Figure 3:**
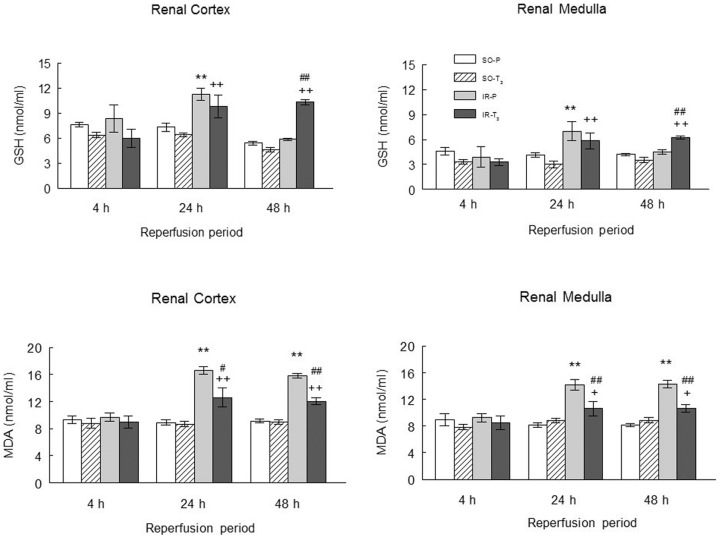
Renal levels of glutathione and MDA in the experimental groups. (n = 10 each group): SO-P =  sham-operated-placebo; SO-T_3_ =  sham-operated, T_3_-treated (100 μg/kg); IR-P =  ischemia-reperfusion, placebo-treated; IR-T_3_ =  ischemia-reperfusion, T_3_-treated. Data are means ± SEM. ** p<0.001 *versus* SO-P group. + p<0.05, ++ p<0.001 *versus* SO-T_3_ group. # p<0.05, ## p<0.001 *versus* IR-P group.

Tissue glutathione levels (in cortex and medulla) were similar in all groups at 4 h of reperfusion, but were increased in the IR-P and IR-T_3_ groups at 24 h and were higher in the IR-T3 group than in the IR-P group or either SO group at 48 h of reperfusion (fig 3). Tissue MDA values (cortex and medulla) were increased in the IR-P group at 24 and 48 h of reperfusion, and this increase was markedly attenuated in the IR-T_3_ group (fig 3).

### Histopathological and immunohistochemical results

**Figure 4:**
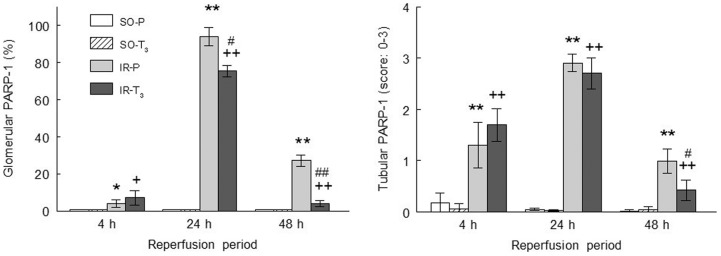
Glomerular (A) and tubular (B) expression of PARP-1 in the experimental groups. (n = 10 each group): SO-P =  sham-operated-placebo; SO-T_3_ =  sham-operated, T_3_-treated (100 μg/kg); IR-P =  ischemia-reperfusion, placebo-treated; IR-T_3_ =  ischemia-reperfusion, T_3_-treated. Data are means ± SEM. * p<0.05, ** p<0.001 *versus* SO-P group.+ p<0.05, ++ p<0.001 *versus* SO-T_3_ group. # p<0.05, ## p<0.001 *versus* IR-P group.

PARP-1 expression was elevated in both IR groups at all reperfusion times, showing the highest levels at 24 h of reperfusion, and it was reduced in the IR-T_3_ group at 24 and 48 h of reperfusion (fig 4).

**Figure 5:**
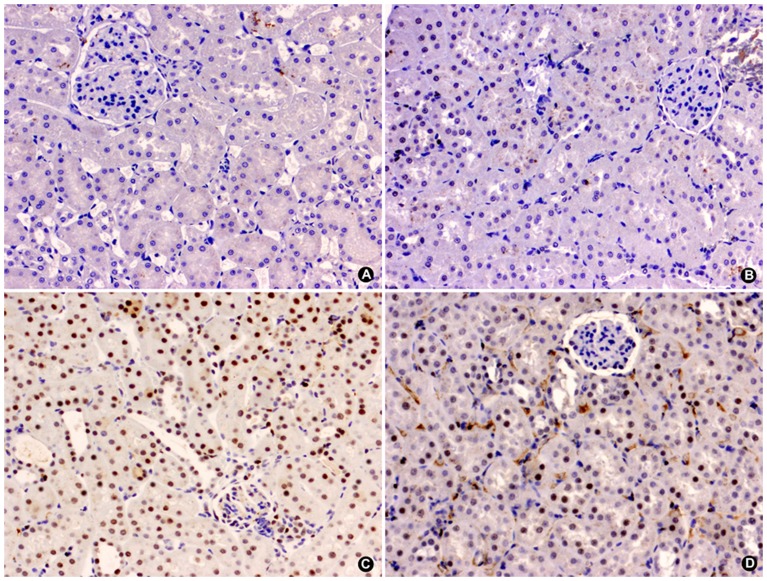
Representative microphotograph of the immunohistochemistry study of PARP-1 expression in renal cortex of male Wistar rats after 48 h of ischemia-reperfusion. Absence of nuclear expression in SO-P (A) and SO-T_3_ (B) groups. The IR-P group (C) shows intense nuclear expression (brown deposit) in >80% of tubular cells. The IR-T3 group (D) shows moderate nuclear expression in <20% of tubular cells (micropolymer peroxidase conjugated, original magnification ×20).

**Figure 6:**
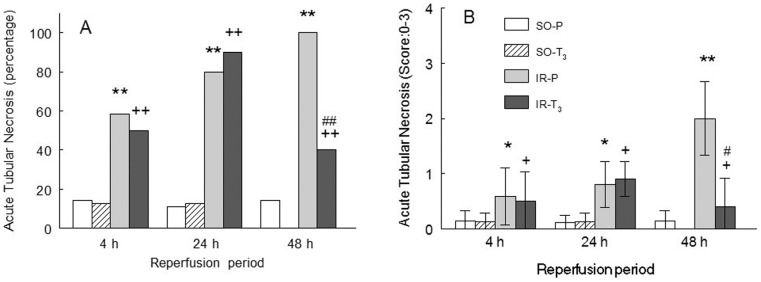
Percentage (A) and score (B) of acute tubular necrosis in the experimental groups. (n = 10 each group): SO-P =  sham-operated-placebo; SO-T_3_ =  sham-operated, T_3_-treated (100 μg/kg); IR-P =  ischemia-reperfusion, placebo-treated; IR-T_3_ =  ischemia-reperfusion, T_3_-treated. Data are means ± SEM. * p<0.05, ** p<0.001 *versus* SO-P group. + p<0.05, ++ p<0.001 *versus* SO-T_3_ group. # p<0.01 *versus* IR-P group.

**Figure 7:**
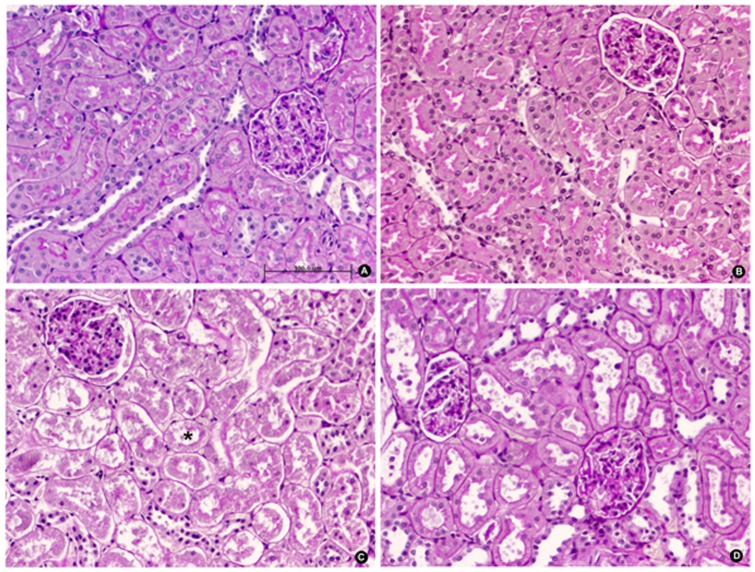
Representative microphotograph of morphological changes in renal parenchyma of male Wistar rats after 48-reperfusion. Absence of glomerular or tubular injury in kidney sections of rats in SO-P (A) and SO-T3 (B) groups. Rats exposed to bilateral renal ischemia-reperfusion pre-treated with placebo, IR-P (C), show intense acute tubular necrosis (asterisk) with severe detachment of epithelial cells from the basement membrane, loss of brush border, and intratubular casts. The IR-T3 group (D) shows mild tubular necrosis and moderate sloughing of tubular cells (PAS, original magnification ×20).

The extent and intensity of tubular nuclear PARP-1 expression concurred with the presence of ATN. PARP-1 was elevated in the IR groups at all reperfusion times and was significantly reduced in T_3_-treated animals at 48 h of reperfusion (fig 4 and 5). The extent and intensity of ATN were directly related to the length of reperfusion time in the IR groups and were significantly reduced in the IR-T_3_ group at 48 h of reperfusion (fig 6 and 7).

At 48 h of reperfusion, regenerative changes were observed in 80% of the rats in the IR-T_3_ group *versus* 20% in the IR group, and cortical and medullar kidney hyperemia was detected in the T_3_-treated groups (43% in each group). The extent of tubular casts was moderate (30%) in the IR-T_3_ group and moderate/severe (60%) in the IR group. No other morphological differences were observed among the four groups.

No differences in tubulointerstitial inflammatory infiltrate (total CD45-positive leukocytes and CD68-positive macrophages) were found among the groups at any reperfusion time (4, 24, or 48 h). The number of myeloperoxidase-positive granulocytes was also similar among the four groups (table 2).

**Table 2:** Assessment of kidney biopsies and quantification of inflammatory infiltrate in rat control and ischemia/reperfusion groups at different times.

Variable	Control 4h	IR- T_3_ 4h	IR-P 4h	Control 24h	IR- T_3_ 24h	IR-P 24h	Control 48h	IR- T_3_ 48h	IR-P 48h
**Capillaritis**	0.0±0.0	0.50±0.5^†^	0.25±0.4	0.0±0.0	0.20±0.4	0.40±0.5*	0.0±0.0	0.50±0.7^†^	0.50±0.5*
**Tubulitis**	0.0±0.0	0.04±0.5	0.08±0.2	0.0±0.0	0.0±0.0	0.10±0.3	0.0±0.0	0.0±0.0	0.10±0.3
**Glomerulitis**	0.0±0.0	0.30±0.4	0.0±0.0	0.0±0.0	0.50±0.5	0.30±0.4*	0.0±0.0	0.0±0.0	0.11±0.3
**CD45/mm^2^**	16.06±5.3	23.99±10.1	21.97±8.3	48.39±18.6	18.55±6.2^†^	11.60±3.6*	21.36±3.3	24.19±9.7	21.97±8.3
**CD68/mm^2^**	12.62±4.4	22.08±14.5	14.26±6.4	41.13±13.9	26.61±13.2	12.90±3.8*	20.96±5.7	32.09±19.5	23.99±9.6
**PMN/mm^2^**	6.08±0.5	8.14±0.8	8.56±0.6	10.40±4.6	12.90±5.1.	12.09±5.5	8.06±3.9	12.72±14.5	15.12±8.1

Values are expressed as mean ± standard deviation.

Control: Placebo-treated (SO-P) + sham-operated T_3_- treated (SO- T_3_); I/R-injured placebo-treated (IR-P); I/R-injured T_3_-treated (IR- T_3_) groups. * P<0.05 IR-P vs. Controls; ^†^ P<0.05 IR- T_3_ vs. Controls; Mann Whitney U-test.

## Discussion

In this study, the administration of a single dose of T_3_ to rats at 24 h before IR significantly diminished the ensuing renal injury, producing clinical and histological improvements and reducing oxidative stress variables, plasma IL6 inflammatory cytokine levels, and the glomerular and tubular expression of PARP-1.

The ARF clinically manifested as an elevation in plasma urea and creatinine levels and was histopathologically evidenced by ATN. Preconditioning with T_3_ attenuated the renal dysfunction, reducing the plasma urea and creatinine levels and increasing the creatinine clearance. Preconditioning with T_3_ was found to have a clear anti-necrotic effect, which can at least in part be explained by the decreased *in situ* expression of tubular PARP-1 and the signs of reduced renal oxidative stress, i.e., decreased tissue (cortex and medulla) MDA values, increased tissue (cortex and medulla) glutathione levels, and decreased urinary isoprostane and hydrogen peroxide levels. These changes diminish the necrosis of tubular epithelial cells, thereby improving clinical renal function.

Our findings are in line with previous observations that preconditioning with T_3_ can protect the liver from I/R injury [14]. The cellular and molecular mechanisms responsible for these salutary effects are not completely understood and are likely to be multifactorial.

Renal preconditioning has been extensively explored as a protective strategy to prevent the consequences of IR injury. It has been produced by subjecting the kidney and other tissues to situations that produce a mild oxidative stress status, such as transient ischemia [22], [23] hyperthermia [24], or hyperbaric oxygenation [25]. T_3_ enhances the O_2_ consumption and ROS production associated with antioxidant depletion, inducing a redox imbalance accompanied by upregulated expression of cytokines [26], superoxide dismutase [27], [28], and anti-apoptotic proteins [27]. These may be adaptive mechanisms to restore redox homeostasis and protect cells from the oxidative stress induced by IR. T_3_ administration may represent a preconditioning stimulus that produces a short-term and reversible redox imbalance, as indicated by the reduced plasma glutathione levels and the tendency to lower renal tissue values in both T_3_-treated groups at 4 h of reperfusion. This effect is devoid of renal toxicity, as demonstrated by the normal renal values in the SO-T3 group.

The main tubular and glomerular damage appears to occur during the post-ischemia reperfusion period, and the generation of ROS has been implicated as a major contributing factor [29], [30]. In our study, T3 preconditioning was associated with a reduction in the oxidative stress components induced by I/R, as evidenced by the decreases observed in plasma, urinary, and tissue levels of oxidative stress variables. The mechanism by which T_3_ attenuates local and systemic oxidative stress may involve the prevention of ROS-dependent oxidative deterioration of biomolecules by re-establishing redox homeostasis. Furthermore, T_3_ may revert the changes in signal transduction and gene expression that underlie I-R-induced kidney injury [29], [31].

Apart from the effects evidenced in the present study, it has been shown [13] that thyroid hormone treatment enhances the recovery of renal ATP and reduces the cell alterations associated with ischemic renal injury. Thus, the beneficial impact of thyroid hormone may also be explained by the activation of renal Na-K-ATPase, given the demonstration by Lo and Edelmann [32] that T_4_ increases not only Na-K-ATPase activity but also the number of Na-K-ATPase units in the renal cortex. Furthermore, other researchers [11] found that T_4_ accelerates reversal of the decline in Na-KATPase activity in several ARF models. Finally, Johnson et al. [12] showed that T_3_ pre-treatment of rabbit proximal tubular cells augments their response to epidermal growth factor (EGF) and increases the number of receptors on renal epithelial cells, accelerating tubular regeneration.

The extent and intensity of glomerular and tubular PARP-1 expression were elevated in the ischemic groups after all reperfusion periods, in agreement with previous reports of an association between renal injury secondary to renal ischemia and PARP-1 overactivation [33], [34]. PARP-1 is a nuclear protein that protects the cell genome by repairing DNA strand breaks [35], catalyzing the ADP-ribosylation of proteins using NAD (+) as substrate [36]. Ischemia-induced PARP-1 over-activation leads to massive NAD + consumption and ATP depletion [37], producing cell necrosis [38]. PARP-1 has been implicated in the pathogenesis of I/R injury in different experimental models. Thus, I/R lesions were reduced by the pharmacological inhibition of PARP-1 in rats [39] and in parp-1 gene knockout mice [40]. In a study in humans, nuclear tubular expression of PARP-1 preceded the morphological features of ATN, and a positive relationship was found between ATN and PARP-1 expression [18]. In the present study, T_3_ treatment reduced PARP-1 expression at 24 and 48 h of reperfusion, likely related to the recovery of renal ATP [13], a mechanism that can contribute to improving I/R renal injury.

As noted in the Introduction section, kidney I/R injury engages both the innate and adaptive immune responses, and several reports have indicated that leukocytes play a role in I/R injury (19–21). Although we found some sporadic differences in the number of total leukocytes (CD45) and macrophages (CD68) detected by immunohistochemistry, there were no differences in the granulocyte count at any reperfusion time. Kidney biopsies from the different groups showed no clusters of inflammatory infiltrate in tubulointerstitium or glomeruli, only the presence of circulating inflammatory cells within vessels. Given the similar granulocyte count to that in the controls, we believe that the beneficial effect of T3 is attributable to its direct impact on renal cells and is not mediated by a reduction in granulocyte count.

In conclusion, preconditioning with T_3_ reduced the clinical and histological signs of renal I/R injury in rats and was associated with reductions in plasma, urinary, and renal oxidative stress variables, plasma IL6 inflammatory cytokine levels, and glomerular and tubular PARP-1 expression. These results suggest an important role for the sequence of oxidative stress, inflammation, and PARP-1 overactivation in the ATN induced by I/R.
